# Factors Influencing Attitude, Safety Behavior, and Knowledge regarding Household Waste Management in Guinea: A Cross-Sectional Study

**DOI:** 10.1155/2016/9305768

**Published:** 2016-03-22

**Authors:** Keita Mamady

**Affiliations:** Department of Epidemiology and Health Statistics, School of Public Health, Central South University, Changsha, Hunan 410078, China

## Abstract

Waste indiscriminate disposal is recognized as an important cause of environmental pollution and is associated with health problems. Safe management and disposal of household waste are an important problem to the capital city of Guinea (Conakry). The objective of this study was to identify socioeconomic and demographic factors associated with practice, knowledge, and safety behavior of family members regarding household waste management and to produce a remedial action plan. I found that no education background, income, and female individuals were independently associated with indiscriminate waste disposal. Unplanned residential area was an additional factor associated with indiscriminate waste disposal. I also found that the community residents had poor knowledge and unsafe behavior in relation to waste management. The promotion of environmental information and public education and implementation of community action programs on disease prevention and health promotion will enhance environmental friendliness and safety of the community.

## 1. Introduction

Humanity continues to develop and produce cutting-edge products in order to fulfill its most fundamental needs of life. However, the resulting production and consumption of resources end up with prominent problems regarding solid waste generation and management in diverse parts of the world [1]. Developed countries' waste disposal practice includes landfilling, composting, incineration, and pyrolysis [2]. Safe management and disposal of household waste are problems that face some metropolitan cities in Guinea [3, 4]. Yet, the environmental pollution associated with indiscriminate waste disposal has serious negative impacts on public health and safety [5, 6].

The major causes of improper management of solid waste are related to the lack of financial management and logistics, deficient municipal infrastructures, lopsided planning pastures, disregard for basic aesthetics, and industrial and commercial growths as well as the perceptions and sociocultural practices [7, 8]. Although inadequate management of solid waste might be attributed to numerous factors, it is essential to emphasize the role of community residents, their attitudes, their waste handling practices, and their interactions with other actors in the waste system because they are the main end-users of waste management facilities [1, 9]. Barrier to solid waste management in Guinea might be quite unique per se in terms of environmental impacts, socioeconomic factors, and cultural heritage, so different areas will find different strategies effective for proper waste management.

Some research studies found that either at-home safety consciousness [9] or knowledge [10] of waste related deleterious health effects is associated with household waste disposal strategy. For example, safety behavior is required to prevent direct contamination and exposure to infectious and injurious substances to health from household waste on the one hand. On the other hand, increasing knowledge can foster positive attitudes and build safe practices among populations. In Guinea, there is a lack of measures aimed at informing the public about the causal connection between environmental pollution and health, and no provision has been made for a long-term evaluation which would make it possible to examine whether the measures are helping to reduce environmentally related health problems in a cost-effective manner [3, 4]. Therefore, research and development in waste management should continue to improve data, models, and concepts related to long-term safety of disposal of long-lived waste.

The main objectives of this study were (1) to identify factors associated with abnormal household waste disposal and (2) to assess the household knowledge of the health and safety risks posed by improper disposal of household special waste.

## 2. Methods

### 2.1. Setting and Population

An interesting aspect to the study is the focus it puts on the largest and most urbanized city of Guinea, Conakry. The city is thought to contain almost a quarter of the population of Guinea. Conakry is a port city on the Atlantic Ocean and serves as the economic, financial, and cultural centre of the country. Its population was estimated in 2014 at 1,667,864 with an area of 450 square kilometers [11]. The city has been experiencing an insufficiency of local waste disposal sites and recycling waste materials [4].

### 2.2. Sample Description

The study sample was a survey of a cross-sectional, multistage, clustered, stratified, weighted design representative of the population of Conakry. The survey was carried out between February and April 2015. As part of the inclusion criteria, respondents were required to (1) be 15 years old or older, (2) freely consent to participate in the survey, (3) speak French or at least one of the Guinean dialects, and (4) suffer from no central nervous system disorder (including Alzheimer's disease, amyotrophic lateral sclerosis (ALS), and behavioral disorders).

The primary sampling unit was the household located within a radius of, at most, 5 km from the major road junctions in the city. Data collectors received assistance from the city planning department to delineate a 5 km radius from each traffic circle and make available the list of major intersections. A random sample of reporting units were drawn proportional to size. The secondary sampling units consisted of random subsampling within the reporting units to obtain a sample of households according to a household list established by the survey team leader and community representatives. The tertiary sampling units were a single member per household, preferably the household head. To obtain the required sample size for this study, a multistage sample design formula was used with margin of error for estimates of the whole population [12]. The combined response rate was 96.0%, for a final sample size of 1093.

### 2.3. Data Collection

To increase the survey response rate, the community leaders were consulted to assist in recruiting data collectors from within their communities. The data collectors were a mix of undergraduates and graduates students of sociology, geography, and medicine. These interviewers were extensively trained with respect to the survey procedures and questionnaire. The data collectors were also specifically trained to ensure that the participants are completely informed of their rights prior to obtaining consent. The survey collected detailed information on respondents at their premises on waste disposal practice. It also asked about knowledge and safety behavior regarding household waste management. The survey questionnaire was constructed by the researchers after an extensive literature search on previous related topics [9, 10]. The reliability coefficient (as assessed by Cronbach's alpha) for this study was excellent, at 0.90, and excellent validity statistics have been previously reported [13, 14].

In this study, three variables are used to represent the outcomes measures: community residents' waste disposal practice, knowledge of community residents concerning the health effect of domestic waste, and safety behavior related to waste handling. The community residents' waste disposal practice derived from the question: “How your community solid waste is often disposed off?” This question consists of three values: municipal accredited dump sites, accredited private sector participation, and open land. These three values were further dichotomized into good waste disposal practice, when the residents dispose of waste directly to the permitted municipal dumpsites or make waste collected by accredited private company from the residents' premises, or poor waste disposal practice, when waste is disposed on the open land. The level of knowledge was defined as “poor” for a score less than 50% and was defined as “good” for a score of 50% and more. The level of safety behavior was defined as “safe” for those scoring higher than the mean score and was defined as “unsafe” for those scoring less than or equal to mean. All covariates collected in the survey were treated as potential confounding and adjusted.

### 2.4. Statistical Procedure

Descriptive analysis was performed to investigate the characteristics of different waste disposal practices of the study population. The multivariate logistic regression analyses were conducted to test the influence of socioeconomic and demographic factors on the community residents' waste disposal practice, their knowledge level of disease causation related to poor waste management, and their safety behavior with regard to waste handling. To assess the likelihood that the respondents will adopt good disposal practice of waste in their community, seven explanatory variables were considered: age, sex, marital status, education attainment, income group, residential area, and distance to permitted dumpsite. To predict the respondents' knowledge of disease causation from waste handling and safety behavior towards waste handling, six predictors were included in the model: age, sex, marital status, education attainment, income group, and residential area. The selection of explanatory variables was based on common sense and literature [1, 15, 16]. For the components of the questionnaire, item and reliability analyses were applied. Currently, the cut-off for statistical significance is set at *P* ≤ 0.05.

## 3. Results

### 3.1. Descriptive Statistics

Table 1 portrays the frequency distribution of socioeconomic and demographic characteristics. There were a total of 1093 respondents. The mean age (± standard deviation) was 39.4 ± 13.3 years, corresponding to an age range from 15 to 71 years. The mean income of the respondents was 372000 Guinean Francs (GNF) (SD ± 299500), ranging from 21000 to 1850000 GNF.

Table 1 also identifies four remarkable methods of waste disposal. Waste disposed in an open land makes up a large proportion (41.4%) followed, respectively, by accredited private sector (24.8%), municipal accredited dumpsites (20.2%), and burning (12.6%). When analyzing waste disposal methods by socioeconomics and demographic aspects, we can quickly identify that the respondents in the age group of 15–24 years often dispose of waste in the open land (39.8%) or by burning (32.1%). The respondents aged 30–39 years either frequently make their waste collected by an accredited private company (45.2%) or discard waste in an open land (33.1%). The most frequent preferred waste disposal method of the respondents in the age group of 40–49 years is open land (56.9%) or municipal accredited dumpsites (30.1%). Respondents aged 50–59 years often throw waste in an open land (56.2%), while those above 60 years of age burn waste in the environment (51.4%). A considerable greater proportion of women (45.6%) discriminately dispose of waste in the open land compared to men (32.5%). Respondents having no education attainment (54.3%) and those with primary school level (47.9%) often dispose of waste in the open land, while the respondents with secondary (36.6%) and tertiary (38.8%) schooling background often favored private companies.

Respondents having an income less than 250001 (59.8%) and income between 250001 and 450000 (38.4%) frequently dispose of waste in an open land, while respondents with income from 450001 to 650000 (68.1%) often dispose of waste in municipal permitted dumpsites. Evidently, the respondents with income between 650001 and 850000 (84.3%) and more than 850000 (43.3%) commonly preferred accredited private sector for waste collection.

While respondents residing in unplanned neighborhood make up a larger percentage in dropping waste in the open land (55.3%), the respondents in planned residential areas appeared to either be affiliated to waste collection companies (47.3%) or take their waste to permitted municipal dumpsites (42.6%). Residents residing less than 50 meters (51.6%) or between 50 and 100 meters (58.4%) away from permitted municipal dumpsites, respectively, dispose of their waste at the accredited municipal dumpsite and make their waste collected by private companies, but the respondents residing at more than 100 meters and over 200 meters away from permitted municipal dumpsites, respectively, discriminately dispose of their waste in the open land (56.5% versus 39.9%) or by burning (15.9% versus 26.0%).

Table 2 illustrates the knowledge of the respondents regarding waste related disease causation. Satisfactorily, 96.2% of the respondents were aware of the fact that reckless handling of waste must be harmful to human health. Unfortunately, 63.7% were unaware of the possible contamination of host such as surface, ground, and piped water at any time as a result of poor waste management. Likewise, 63.4% of the respondents believe that poor disposal of children's feces has no adverse health effects. Concerning the evaluation of the respondents about their knowledge of the diseases caused by poor management of waste, 65.8%, 63.2%, and 52.1% of the respondents are conscious that, respectively, typhoid, cholera, and malaria can result from poor waste handling. A very few people believe that diarrhea (23.9%), dysentery (5.2%), respiratory infection (4.0%), and injury (1.5%) might be the result of poor waste handling.

Table 2 delineates the safety behavior of the respondents in relation to waste management. Interestingly, 51.6% of the respondents adopt an important aspect of personal hygiene; that is, they properly wash their hand after waste disposal. Importantly, 50.2%, 55.1%, and 72.5% of the respondents, respectively, throw garbage daily, usually keep garbage near the outside door, and currently sleep under a mosquito net. Other aspects of safety behavior are nevertheless cause of concern; the respondents do not prevent their children from playing near the solid waste (73.8%), they leave the garbage unprotected near the outside door (73.3%), they do not often wash the rubbish container with soap and water or clean with dry earth or sand (89.7%), and they do not usually treat water from unprotected and suspicious surface, ground, and piped sources before use (86.6%).

### 3.2. Household Waste Management Practice

In Table 3, the logistic regression model showed that the variables such as sex, education attainment, marital status, household income, residential area, and the distance of the respondents away from the permitted dumpsite made a statistically independent contribution to the model. The strongest and isolate predictors of poor waste disposal practice were residential area, education attainment, and sex with respective odd ratios of 5.81, 3.02, and 2.50. Odd ratio for income indicates little change in the likelihood of poor waste disposal. People who are residing 50 meters away from municipal permitted dumpsites were less likely to poorly dispose of waste with an odd ratio of 0.04 (Table 3).

### 3.3. Household Knowledge of Waste Related Disease Causation

From Table 4, the logistic regression model showed that only sex, education attainment, and income made significant contributions to prediction. The odds of a woman being knowledgeable of the health effects related to waste mismanagement were 0.59 times lesser than the odds for man. In the same breath, the respondents having no education, primary education, and secondary education were, respectively, less likely to know the implication of waste in disease causation. The odd ratio value indicates that the respondents of at least a disposable income between 450001 and 650000 Guinean Francs are 5.10 times more likely to know the role of waste in disease causation (Table 4).

### 3.4. Household Waste Handling Safety Behavior

The result of logistic regression analysis to appraise the influence of a set of factors on the likelihood that the respondent would adopt safety behavior related to waste handling is presented in Table 5. Considering the full model, age, sex, education attainment, and income made a unique statistically significant contribution to the model. The strongest predictor of having safe behavior was being aged between 15 and 39 years which had an odd ratio of 4.21. The respondents having female gender, no education, and income less than 250001 were less likely to adopt safe behavior (Table 5).

## 4. Discussion

This study is not only the first to develop standardized and sustainable approaches that identify broad spectrum of safety and knowledge-based variables but also the first to predict and then directly test the effects of socioeconomic and demographic factors on waste related safety and knowledge. The results of this study provide a real support for the hypothesis that the household has important roles and responsibilities in indiscriminate dumping of municipal waste. The overall proportion of community residents who adopt adequate waste disposal practice was 78.3% versus 92.0% for residents who inadequately dispose of waste. The predictors of poor waste disposal practice are residential area, education attainment, sex, income, and residence at 50 meters away from municipal permitted dumpsites. Similar findings have been reported in previous research [15–17].

In the multivariate analysis, the strongest predictor of indiscriminate disposal of waste was unplanned residential area, as evidenced by the adjusted odd ratio, 5.81. This echoes the other major finding that indiscriminate waste disposal alongside with inadequate waste collection is strongly linked with the existence of unplanned settlements in the city [15]. Our finding seems to typically reflect the context of the study area, where the strategy to promote urban sustainability through the implementation of management and planning process is inadequate [18, 19]. Authorities should be encouraged to promote environmental information and education of the public, which will also undoubtedly be in the authorities' own interests, in that the extent to which people participate effectively, particularly women, can only improve through education. If waste is collected by private corporations, the cost should be designed to meet the affordability of low-income people. A responsible common effort to refurbish existing road networks in poor state and to build good paved road networks in the city and suburbs connecting all other settlements will ensure the success of waste management in the city.

Another important goal of this piece of research was to assess the community's knowledge of the health risk of improper waste management. There is ample evidence that if the community residents have immense knowledge of the harmful effect of poor waste management in general, they have a very little knowledge of the implication of waste in environmental contamination and transmission. It should be also noted that most respondents are aware that improper management of waste leads to cholera, typhoid, and malaria. However, there was a lack of understanding about some economically important diseases such as dysentery, diarrhea, respiratory infection, and injury. The poor knowledge level of the respondents was strongly and independently influenced by income, education, and sex, indicating that more effort is needed to adopt community action programs on disease prevention and health promotion with particular focus on women. For economically disadvantaged household that cannot easily have access to mass media, great outreach programs should be provided for information dissemination.

This present study has demonstrated that the respondents are used to adopting rudimentary safety measures with regard to waste management such as proper washing of hands after waste disposal, throwing garbage daily, keeping garbage near the outside door, or sleeping under a mosquito net. Unfortunately, the respondents lack one of the most adequate safety behaviors that could be relevant in breaking the chain of contamination from noxious substances and harmful microbial and viral transmissions. That is to say, only a small number of respondents usually use treated water from unprotected and suspicious surface, ground, and piped sources. In general, the inadequate safety behavior is independently associated with age, sex, education attainment, and household income of the respondents. In response to these challenging circumstances, the Guinean government has to seek more assistance from the development partners to avail itself of financial support as well as much-valued technical assistance and advice to improve the delivery of community-based health education. To make the management of such investment efficient and effective, the Guinean government can make all effort to ensure the availability of responsible human resources that are respectful of the community rights.

The main strengths of this study are the following: it used the large sample size with three outcome measures, accounted for confounding factors, and established good survey reporting method. Interestingly, this study can address the need for comprehensive information and tools to assist policy makers and stakeholders in adjusting current programs and planning future programs. For health educators, the study will better promote healthy handling of household waste to diverse populations. And, for researchers, this study will contribute towards the improvement of data comparability.

## 5. Conclusion

This study provides evidence that household and community groups' waste disposal practice is careless with the environment. Such waste disposal practice with disregard for the possible environmental consequences is possibly influenced by specific socioeconomic status (sex, education attainment, and household income) and geographic risk factors (residential area and residents' distance to municipal permitted dumpsite). It demonstrated that the respondents not only have poor knowledge of the adverse health effect with regard to improper waste handling but also have unsafe behavior towards safety practices. This research suggests that the promotion of environmental information and education of the public and adoption of community action programs on disease prevention and health promotion will enhance comfort, environmental friendliness, and safety of the community. The government could create an environment where innovation and the promotion of knowledge can flourish. Investments in knowledge and innovation are keys to improving the country's productivity performance and increasing the community's standard of living. Future study should focus on the financial role of the government and/or the management efforts of waste collection corporations.

## Figures and Tables

**Table 1:** Socioeconomic and demographic characteristics and solid waste disposal methods of the respondents (*N* = 1093).

Variables	Frequency (%)	Waste disposal methods			
		MAD^*∗*^	APD^*∗*^	Open land	Burning
		*N* (%)	*N* (%)	*N* (%)	*N* (%)
Overall	1093 (100%)	221 (20.2%)	271 (24.8%)	452 (41.4%)	149 (13.6%)
Age group					
15–39	641 (58.6%)	89 (13.9%)	216 (33.7%)	227 (35.4%)	109 (17.0%)
40–59	378 (34.6%)	113 (29.9%)	49 (13.0%)	214 (56.6%)	2 (0.5%)
≥60	74 (6.8%)	19 (25.7%)	6 (8.1%)	11 (14.9%)	38 (51.4%)
Sex					
Male	351 (32.1%)	104 (29.6%)	93 (26.5%)	114 (32.5%)	40 (11.4%)
Female	742 (67.9%)	117 (15.8%)	178 (24.0)	338 (45.6)	109 (14.7)
Marital status					
Single	210 (19.2%)	41 (19.5%)	55 (26.2%)	80 (38.1%)	34 (16.2%)
Married	742 (67.9%)	151 (20.4%)	179 (24.1%)	313 (42.2%)	99 (13.3%)
Divorced	54 (4.9%)	16 (29.6%)	18 (33.3%)	15 (27.8%)	5 (9.3%)
Widowed	87 (8.0%)	13 (14.9%)	19 (21.8%)	44 (50.6%)	11 (12.6%)
Education attainment					
None	565 (51.7%)	93 (16.5%)	88 (15.6%)	307 (54.3%)	77 (13.6%)
Primary	140 (12.8%)	19 (13.6%)	33 (23.6%)	67 (47.9%)	21 (15.0%)
Secondary	308 (28.2%)	74 (24.0%)	119 (36.6%)	66 (21.4%)	49 (15.9%)
Tertiary	80 (7.3%)	35 (43.8%)	31 (38.8%)	12 (15.0%)	2 (2.5%)
Household income					
Less than 250001	639 (58.5%)	71 (11.1%)	47 (7.4%)	382 (59.8%)	139 (21.8%)
250001 to 450000	146 (13.4%)	37 (25.3%)	51 (34.9%)	56 (38.4%)	2 (1.4%)
450001 to 650000	91 (8.3%)	62 (68.1%)	27 (29.7%)	2 (2.2%)	0
650001 to 850000	127 (11.6%)	19 (15.0%)	107 (84.3%)	1 (0.8%)	0
More than 850000	90 (8.2%)	32 (35.6%)	39 (43.3%)	11 (12.2%)	8 (8.9%)
Residential area					
Unplanned residential area	776 (71.0%)	86 (11.1%)	121 (15.6%)	429 (55.3%)	140 (18.0%)
Planned residential area	317 (29.0%)	135 (42.6%)	150 (47.3%)	23 (7.3%)	9 (2.8%)
Distance to permitted dumpsite					
Less than 50 meters	62 (5.7%)	32 (51.6%)	26 (41.9%)	3 (4.8%)	1 (1.6%)
Between 50 and 100 meters	231 (21.1%)	67 (29.0%)	135 (58.4%)	26 (11.3%)	3 (1.3%)
More than 100 meters	627 (57.4%)	98 (15.6%)	75 (12.0%)	354 (56.5%)	100 (15.9%)
Over 200 meters	173 (15.8%)	24 (13.9%)	35 (20.2%)	69 (39.9%)	45 (26.0%)

^*∗*^MAD: municipal accredited dumpsites; APS: accredited private sector.

**Table 2:** Knowledge of the health effects and safety behavior of the respondents regarding waste handling (*N* = 1093).

Questions with correct responses	*N* (%) correct responses	95% CI
*Knowledge*		
Is poor waste disposal harmful? (Yes)	1052 (96.2%)	[0.950, 0.972]
Can surface water/ground water/piped water be contaminated at any time? (Yes)	397 (36.3%)	[0.335, 0.392]
Are children's feces as dangerous as those of adults? (Yes)	400 (36.6%)	[0.338, 0.395]
Are these following diseases related to poor waste disposal?		[0.603, 0.660]
Cholera (yes)	691 (63.2%)	[0.603, 0.660]
Typhoid (yes)	719 (65.8%)	[0.629, 0.685]
Dysentery (yes)	57 (5.2%)	[0.041, 0.067]
Malaria (yes)	569 (52.1%)	[0.491, 0.550]
Diarrhea (yes)	261 (23.9%)	[0.215, 0.265]
Injury (yes)	16 (1.5%)	[0.009, 0.024]
Respiratory infection (yes)	44 (4.0%)	[0.030, 0.054]

*Safety behavior*		
Do your children play near the solid waste? (No)	286 (26.2%)	[0.237, 0.289]
Do you buy any food from shops near solid waste? (No)	521 (47.7%)	[0.447, 0.506]
Do you properly wash your hands after waste disposal? (Yes)	564 (51.6%)	[0.486, 0.546]
Do you drink boiled water? (Yes)	97 (8.9%)	[0.073, 0.107]
Do you throw garbage daily? (Yes)	549 (50.2%)	[0.473, 0.532]
Do you usually keep garbage near the outside door? (No)	602 (55.1%)	[0.521, 0.580]
Do you leave the garbage unprotected near the outside door? (No)	292 (26.7%)	[0.242, 0.294]
Do you allow the rubbish container to overflow? (No)	526 (48.1%)	[0.452, 0.511]
Do you wash the rubbish container with soap and water or clean with dry earth or sand? (Yes)	113 (10.3%)	[0.087, 0.123]
Are children feces thrown away with other household waste? (No)	477 (43.6%)	[0.407, 0.466]
Do you usually treat water from unprotected and suspicious surface, ground, and piped sources before use? (Yes)	125 (11.4%)	[0.097, 0.135]
Do you sleep under a mosquito net? (Yes)	792 (72.5%)	[0.697, 0.750]

**Table 3:** Binary logistic regression model of association between solid waste disposal methods and socioeconomic and demographic characteristics of residents (*N* = 1093).

Characteristics	Good disposal practice *N* (%)	Unadjusted		Adjusted	
		OR (95% CI)	*P* value	OR (95% CI)	*P* value
Age					
15–39	305 (47.6%)	0.56 (0.34–0.93)	0.03	0.46 (0.20–1.07)	0.07
40–59	162 (42.9%)	0.68 (0.40–1.15)	0.15	0.65 (0.32–1.33)	0.24
≥60	25 (33.8%)	Reference		Reference	
Sex					
Female	295 (39.8%)	1.94 (1.50–2.51)	0.00	2.50 (1.46–4.28)	0.00
Male	197 (56.1%)	Reference		Reference	
Marital status					
Single	96 (45.7%)	0.69 (0.41–1.16)	0.16	0.63 (0.27–1.46)	0.28
Married	330 (44.5%)	0.73 (0.46–1.15)	0.17	0.77 (0.36–1.65)	0.50
Divorced	34 (63.0%)	0.34 (0.17–0.69)	0.00	0.28 (0.10–0.82)	0.02
Widowed	32 (36.8%)	Reference		Reference	
Education attainment					
None	181 (32.0%)	10.0 (5.47–18.28)	0.00	3.02 (1.26–7.20)	0.01
Primary	52 (37.1%)	7.98 (4.08–15.61)	0.00	2.19 (0.82–5.84)	0.12
Secondary	193 (62.7%)	2.81 (1.51–5.23)	0.00	1.59 (0.63–4.01)	0.33
Tertiary	66 (82.5%)	Reference		Reference	
Household income					
Less than 250001	118 (18.5%)	16.50 (9.57–28.43)	0.00	1.44 (0.63–3.26)	0.00
250001 to 450000	88 (60.3%)	2.46 (1.35–4.51)	0.00	0.27 (0.11–0.64)	0.00
450001 to 650000	89 (97.8%)	0.08 (0.02–0.37)	0.00	0.02 (0.01–0.12)	0.00
650001 to 850000	126 (99.2%)	0.03 (0.0– 0.23)	0.00	0.02 (0.00–0.19)	0.00
More than 850000	71 (78.9%)	Reference		Reference	
Residential area					
Unplanned residential area	207 (26.7%)	24.48 (16.43–36.47)	0.00	5.81 (3.25–10.38)	0.00
Planned residential area	285 (89.9%)	Reference		Reference	
Distance to permitted dumpsite					
Less than 50 m	58 (93.5%)	0.04 (0.12–0.10)	0.00	0.16 (0.05–0.57)	0.00
Between 50 and 100 m	202 (87.4%)	0.07 (0.05–0.12)	0.00	0.72 (0.35–1.45)	0.35
More than 100–200 m	173 (27.6%)	1.36 (0.95–1.96)	0.10	1.91 (1.15–3.17)	0.01
Over 200 meters	59 (34.1%)	Reference		Reference	
*x* ^2^				697.471	
df				14	
%				85.8	

^*∗*^ *P* < 0.05.

**Table 4:** Binary logistic regression model of association between knowledge level of the respondents regarding waste related disease causation and socioeconomic and demographic characteristics of residents (*N* = 1093).

Characteristics	Good knowledge *N* (%)	Unadjusted		Adjusted	
		OR (95% CI)	*P* value	OR (95% CI)	*P* value
Age					
15–39	247 (38.5%)	1.69 (0.99–2.90)	0.06	0.80 (0.40–1.61)	0.53
40–59	142 (37.6%)	1.63 (0.93–2.83)	0.09	0.83 (0.46–1.51)	0.54
≥60	20 (27.0%)	Reference		Reference	
Sex					
Female	246 (33.2%)	0.57 (0.44–0.74)	0.00	0.59 (0.39–0.89)	0.01
Male	163 (46.4%)	Reference		Reference	
Marital status					
Single	86 (41.0%)	1.39 (0.82–2.34)	0.22	1.33 (0.71–2.49)	0.38
Married	281 (37.9%)	1.22 (0.76–1.95)	0.41	1.65 (0.93–2.95)	0.09
Divorced	13 (24.1%)	0.63 (0.30–1.37)	0.24	0.44 (0.18–1.06)	0.07
Widowed	29 (33.3%)	Reference		Reference	
Education attainment					
None	118 (20.9%)	0.10 (0.06–0.17)	0.00	0.08 (0.04–0.15)	0.00
Primary	35 (25.0%)	0.13 (0.07–0.24)	0.00	0.11 (0.06–0.23)	0.00
Secondary	198 (64.3%)	0.68 (0.40–1.18)	0.00	0.66 (0.35–1.22)	0.10
Tertiary	58 (72.5%)	Reference		Reference	
Household income					
Less than 250001	178 (27.9%)	0.51 (032–0.79)	0.00	1.26 (0.66–2.42)	0.49
250001 to 450000	57 (39.0%)	0.84 (0.49–1.43)	0.56	2.07 (1.01–4.26)	0.05
450001 to 650000	64 (70.3%)	3.10 (1.68–5.72)	0.00	5.10 (2.44–10.66)	0.00
650001 to 850000	71 (55.9%)	1.66 (0.96–2.86)	0.07	1.46 (0.76–2.81)	0.25
More than 850000	39 (56.7%)	Reference		Reference	
Residential area					
Unplanned residential area	237 (30.5%)	0.37 (0.28–0.49)	0.00	0.83 (0.53–1.30)	0.41
Planned residential area	172 (54.3%)	Reference		Reference	
*x* ^2^				290.863	
df				14	
%				76.7	

^*∗*^ *P* < 0.05.

**Table 5:** The impact of socioeconomic and demographic factors on safety behavior of respondents regarding waste handling (*N* = 1093).

Characteristics	Safety behavior *N* (%)	Unadjusted		Adjusted	
		OR (95% CI)	*P* value	OR (95% CI)	*P* value
Age					
15–39	306 (47.7%)	2.84 (1.63–4.94)	0.00	4.21 (1.96–9.02)	0.00
40–59	135 (35.7%)	1.73 (0.98–3.06)	0.06	1.40 (0.75–2.61)	0.30
≥60	18 (24.3%)	Reference		Reference	
Sex					
Female	258 (34.8%)	0.40 (0.31–0.52)	0.00	0.176 (0.11–0.28)	0.00
Male	201 (57.3%)	Reference		Reference	
Marital status					
Single	107 (51.0%)	1.88 (1.12–3.14)	0.02	0.96 (0.52–1.79)	0.91
Married	291 (39.2%)	1.17 (0.73–1.85)	0.52	0.96 (0.55–1.70)	0.89
Divorced	30 (55.6%)	2.26 (1.13–5.52)	0.02	1.09 (0.49–2.46)	0.83
Widowed	31 (35.6%)	Reference		Reference	
Education attainment					
None	136 (24.1%)	0.17 (0.10–0.28)	0.00	0.20 (0.11–0.37)	0.00
Primary	74 (52.9%)	0.60 (0.34–1.06)	0.08	0.37 (0.19–0.74)	0.01
Secondary	197 (64.0%)	0.96 (0.57–1.60)	0.86	0.85 (0.45–1.58)	0.60
Tertiary	52 (65.0%)	Reference		Reference	
Household income					
Less than 250001	240 (37.6%)	0.29 (0.18–0.46)	0.00	0.28 (0.14–0.56)	0.00
250001 to 450000	49 (33.6%)	0.24 (0.14–0.42)	0.00	0.31 (0.15–0.66)	0.00
450001 to 650000	25 (27.5%)	0.18 (0.10–0.34)	0.00	0.22 (0.11–0.46)	0.00
650001 to 850000	84 (66.1%)	0.93 (0.52–1.65)	0.80	0.53 (0.28–1.02)	0.06
More than 850000	61 (67.8%)	Reference		Reference	
Residential area					
Unplanned residential area	314 (40.5%)	0.81 (0.62–1.05)	0.11	2.30 (1.37–3.85)	0.00
Planned residential area	145 (45.7%)	Reference		Reference	
*x* ^2^				277.409	
df				13	
%				72.1	

Score mean ± SD = 4.54 ± 1.87.

^*∗*^ *P* < 0.05.
